# Supplementary material for: Splice-Junction-Based Mapping of Alternative Isoforms in the Human Proteome
Source: Cell Rep. Author manuscript; Available in PMC 2020 Jan 15. (PMC6961840; doi:10.1016/j.celrep.2019.11.026)
Supplement: 2 [file NIHMS1546469-supplement-2.zip › DF1/PXD009737/TestisTrypsin/TestisTrypsin_49_PDZD2_PTVLLGSSAVIR.pdf]

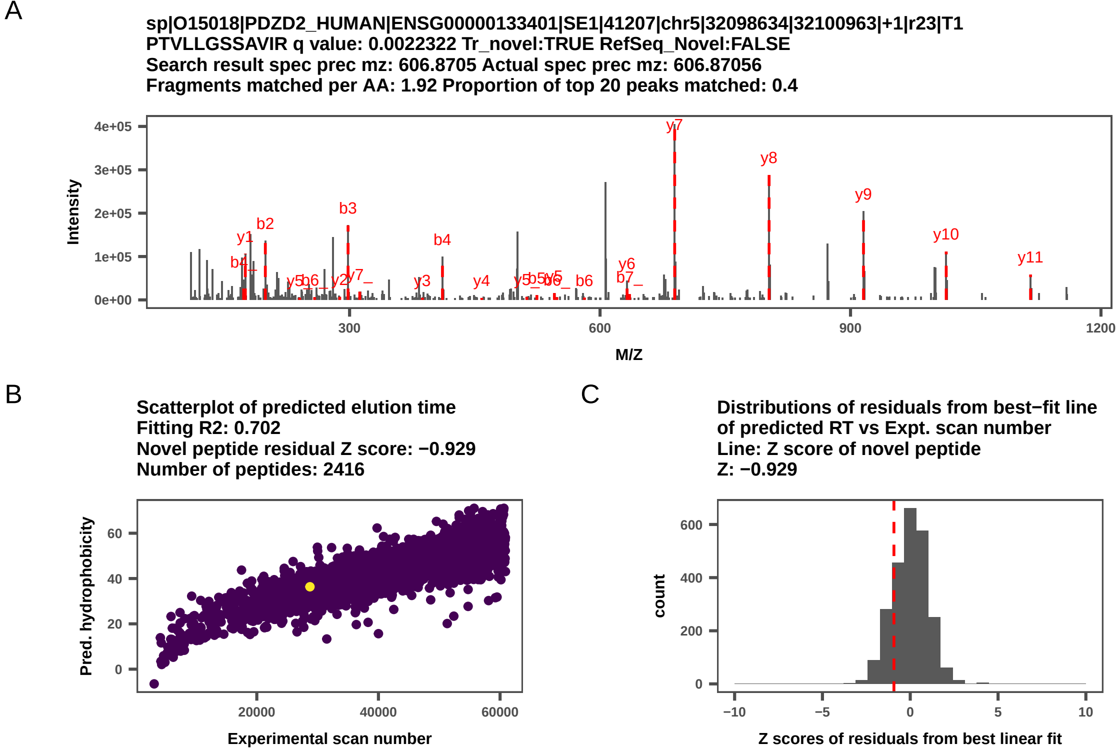
A
sp|O15018|PDZD2_HUMAN|ENSG00000133401|SE1|41207|chr5|32098634|32100963|+1|r23|T1
PTVLLGSSAVIR q value: 0.0022322 Tr_novel:TRUE RefSeq_Novel:FALSE
Search result spec prec mz: 606.8705 Actual spec prec mz: 606.87056
Fragments matched per AA: 1.92 Proportion of top 20 peaks matched: 0.4
Intensity
4e+05
3e+05
2e+05
1e+05
0e+00
y7
y8
y9
b3
b2
y1
b4
y10
y11
y6
b7
y3
y4
b6
300
600
900
1200
M/Z
B
Scatterplot of predicted elution time
Fitting R2: 0.702
Novel peptide residual Z score: −0.929
Number of peptides: 2416
Pred. hydrophobicity
60
40
20
0
20000
40000
60000
Experimental scan number
C
Distributions of residuals from best−fit line
of predicted RT vs Expt. scan number
Line: Z score of novel peptide
Z: −0.929
count
600
400
200
0
−10
−5
0
5
10
Z scores of residuals from best linear fit
